# Genetic and Pathogenic Characteristics of an Emerging Highly Virulent Recombinant Lineage Korean Clade C PRRSV Strain

**DOI:** 10.1155/2024/5785557

**Published:** 2024-08-12

**Authors:** Jeongmin Suh, Chanhee Chae

**Affiliations:** Department of Veterinary Pathology, College of Veterinary Medicine, Seoul National University, Seoul 08826, Republic of Korea

## Abstract

A strain of porcine reproductive and respiratory syndrome virus (PRRSV) was isolated from lung tissue of a pig showing severe respiratory clinical signs from a farm in Gyeongsang province of South Korea. This PRRSV strain, designated as SNUVR220803, was classified within the lineage Korean clade C (LKC) based on a phylogenetic analysis of the ORF5 gene. A whole-genome analysis was conducted on the SNUVR220803 strain, which appears to be a recombinant between the PRRSV strains K07-2273 (part of LKC lineage) and Ingelvac MLV (part of Lineage 5). The Nsp2 amino acid sequence of this strain features a deletion of four additional amino acids, setting it apart from the typical Korean clades A, B, and C lineages. An animal inoculation experiment was conducted with 24 pigs divided into three groups: 12 pigs in the inoculated group, six in the sentinel group, and six in the negative control group. Inoculated pigs exhibited persisting hyperthermia (≥40.3°C) for 5 days, palpebral edema, and cyanosis. Subsequently, these pigs suffered from severe respiratory distress and cachexia, leading to a mortality rate of 58.3% (7 out of 12 pigs) at 14 days postinoculation (dpi). Body weight decreased post-SNUVR220803 strain infection in both the inoculated and sentinel groups. Gross pathology revealed noncollapsed lungs and serous effusion in the pericardial and peritoneal cavities. Microscopic analysis revealed severe interstitial pneumonia, while immunohistochemistry confirmed the presence of PRRSV antigen in the lungs, lymph nodes, thymus, kidneys, and the heart. Additionally, the levels of cytokines such as tumor necrosis factor-*α* (TNF-*α*), interferon-*α* (IFN-*α*), and IL-10 were significantly elevated in the plasma of infected pigs. These observations indicate that the LKC recombinant strain, combined with Lineage 5, possesses high virulence and infectivity as characterized by distinctive exudative lesions.

## 1. Introduction

Porcine reproductive and respiratory syndrome virus (PRRSV) continues to be a leading cause of severe economic loss within the global swine industry and presents significant health challenges for swine production. Porcine reproductive and respiratory syndrome (PRRS) is acknowledged as a critical health issue in swine that leads to reproductive failures in pregnant sows as well as respiratory complications in pigs across all age groups. It falls under the Arteriviridae family within the order of Nidovirales, possessing an envelope that encapsulates a positive-sense, single-stranded RNA [1]. PRRSV is bifurcated into two main genotypes: Type 1 (PRRSV-1), previously referred to as the European genotype, and the Type 2 (PRRSV-2), formerly known as the North American genotype [2]. A classification approach based on phylogenetic relationships has been suggested for PRRSV, which organizes PRRSV-1 strains into three subtypes, Subtype 1 (Lelystad virus-like), Subtype 2, and Subtype 3 (Lena-like), and PRRSV-2 into nine lineages (Lineage 1–9) [3, 4].

Based on the genetic analysis of the ORF5 region, PRRSV-2 has been organized into nine lineages, spanning Lineage 1 to 9 [4]. South Korea has also isolated additional distinct PRRSV-2 strains that do not belong to Lineages 1–9 and has designated them as lineage Korean A (LKA), B (LKB), and C (LKC) [5, 6, 7]. Regarding these Korean lineages, LKA was first reported in 2003 [8], while LKB and LKC were first identified in 2014 and 2005, respectively [5]. PRRSV-2 Lineage 1 was the most prevalent (25/62, 40.32%) of the lineages in South Korea, followed by LKC (13/62, 20.97%), LKB (10/62, 16.13%), Lineage 5 (9/62, 14.52%), and LKA (5/62, 8.06%) [6]. Therefore, within Korean swine populations, the lineage Korean clades (LKA, LKB, and LKC) are predominantly observed; however, the precise origins of country-specific lineages remain unidentified [5, 9].

In 2021–2022, PRRSV caused devastating reproductive failure in sows and respiratory distress in growing pigs within southeastern (Gyeongsang province) Korean farms. Diagnostic cases from one farm were submitted to the Veterinary Diagnostic Laboratory at Seoul National University, where this epidemic field PRRSV strain was identified as LKC and named as SNUVR220803. A preceding study evaluated an LKC PRRSV strain isolated in year 2013 (CA-2 strain) [10]. Pigs that were experimentally infected with the CA-2 strain had higher rectal temperatures than those from the negative control group at 4 days postinoculation (dpi) and 11 dpi. Macroscopic lung lesions were not significantly different between the CA-2 strain-infected group and negative control group [10]. In contrast, LKC PRRSV (SNUVR220803 strain) was isolated in 2022 from Korean herds during the devastating outbreak of PRRS. The objective of this study was to investigate the genetic and pathogenic characterization of the epidemic LKC PRRSV (SNUVR220803 strain) isolated from a grower pig exhibiting symptoms of a respiratory condition linked to an infection by PRRSV.

## 2. Materials and Methods

### 2.1. Farm History

In January 2022, a farm with 1,200 sows reported an ongoing problem with PRRS. In the observed cases, pregnant sows experienced a high incidence of abortions and stillbirths, while piglets were afflicted with respiratory issues. This resulted in a case fatality rate ranging between 30% and 50% and a decrease in the average number of live-born piglets per sow from 12.5 to 7.43. Additionally, there was a noticeable rise in the number of stillborn piglets (increased from 3% to 20%), mummified fetuses (from 2.2% to 33.5%), and preweaning mortality rates (from 9.2% to 67.8%) in field observations. The duration of the disease in growing pigs spanned from 5 to 20 days, with the first mortality documented on the 7th day following the onset of monitoring. Symptoms observed during the outbreaks included severe fever, coughing, loss of appetite, reddening of the skin, and cyanosis of the ears, with nearly all exposed pigs becoming ill and a significant number succumbing to the disease. The outbreak continued for several months, showing no signs of abatement, which can be attributed to the initial lack of understanding of the causative agent and the ineffectiveness of treatment protocols. The PRRSV, hereafter named SNUVR220803, was isolated from necropsy samples of a grower pig afflicted with a respiratory disease linked to infection by PRRSV.

### 2.2. Virus Isolation and Propagation

Clinical samples were collected from pigs at the farm described above, including blood samples, lung tissue, and inguinal lymph nodes. Plasma was separated from the blood samples by centrifugation at 2,500 rpm for 5 min. Tissue samples were homogenized using a Stomacher for 1 min, followed by a freeze–thaw cycle in a −80°C deep freezer. Lysates from the tissue samples were centrifuged at 3,000 rpm for 5 min to obtain the supernatant, which was then filtered through a 0.22-*µ*m pore size syringe filter for virus isolation and genomic analysis.

MARC-145 cells were cultured at 37°C in a 5% CO_2_ incubator using Dulbecco's Modified Eagle's Medium (DMEM) supplemented with 10% fetal bovine serum (FBS) and 1% penicillin–streptomycin. MARC-145 cultures with 80% confluency were inoculated with the filtered lysates diluted 1 : 2 with DMEM and incubated for 3 hr. The cultures were then washed with Dulbecco's phosphate buffered saline (D-PBS) containing magnesium and calcium and incubated for 4 days. A cell culture inoculated with lung homogenate demonstrated a cytopathic effect and underwent the plaque purification method described by Liu et al. [11]. Briefly, virus plaques in the MARC-145 culture were picked using sterile glass Pasteur pipettes and propagated in additional MARC-145 cell cultures.

The purified PRRSV-infected cell culture media and harvested cells were stored in −80°C deep freezer, and a freeze–thaw cycle was performed. Tests for potential contamination by pathogens, including porcine parvovirus, porcine circovirus Types 2 and 3, classical swine fever virus, porcine respiratory coronavirus, swine influenza A (H1N1 and H3N2) viruses, and *Mycoplasma hyopneumoniae*, were conducted using real-time PCR. None of these pathogens were detected in the cell cultures at the DNA and RNA levels. A 0.22-*µ*m pore size filtrate from the third passage of the isolated virus was used for inoculation in an in vivo experiment.

### 2.3. Immunofluorescence Assay (IFA), Plaque Assay, and Virus Titration

Pulmonary alveolar macrophages (PAM) were obtained through bronchoalveolar lavage (BAL) using PBS with 1% Gibco™ antibiotic–antimycotic (Thermo Fisher Scientific, Waltham, MA) from a 3-week-old PRRSV-free piglet. PAM cells were cultured in Roswell Park Memorial Institute (RPMI) media with 10% FBS, 1% penicillin–streptomycin, 0.2% gentamicin, and 0.5% amphotericin B. They were then inoculated with the 3^rd^ passage of the isolated SNUVR220803 virus at a multiplicity of infection (MOI) of one for 1 hr, except for cells mock-inoculated with sterile RPMI medium. After a 2-day incubation, the PAM cells were fixed with 4% paraformaldehyde for 15 min, permeabilized with 0.5% Triton X-100 in PBS buffer for 5 min at room temperature (RT), and incubated with 1 : 1,000 dilution of PRRSV nucleocapsid-specific monoclonal antibody (SR30, Rural Technologies Inc., Brookings, SD). After washing three times, the PAM cells were treated with 10-*µ*g/mL Donkey anti-mouse IgG (H + L) highly cross-adsorbed secondary antibody, Alexa Fluor™ 488 (Thermo Fisher Scientific, Waltham, MA) for 1 hr at RT. Following a 5-min nuclear counterstaining with 4′,6-diamidino-2-phenylindole (DAPI), the stained cells were observed using an LSM700 confocal microscope (Carl Zeiss, Gottingen, Germany).

The plaque assay was performed using MARC-145 cells inoculated with a tenfold diluted SNUVR220803 virus for 1 hr in six-well plates. The inoculated cells were overlaid with 1% SeaPlaque™ Agarose (Thermo Fisher Scientific, Waltham, MA) in DMEM supplemented with 10% FBS and 1% antibiotic–antimycotic, followed by a 4-day incubation at 37°C in a 5% CO_2_ incubator. The cells were fixed with 4% formaldehyde for 1 hr, and then, the agarose plugs were removed. They were stained with 0.5% crystal violet in PBS for 5 min and washed with tap water.

MARC-145 cells in monolayer were inoculated with each of SNUVR220803, another LKC SNUVR240314 strain, and RespPRRS_MLV at 0.01 MOI in six-well plates for 1 hr. The supernatant was collected at time points from 1 to 6 days after inoculation for virus titration. The collected supernatant was used for TCID_50_/mL calculation in 96-well plates.

### 2.4. Whole-Genome Sequencing

The Direct-zol RNA Miniprep kit (Zymo Research, Irvine, CA) was used to extract RNA from virus in cell culture lysates for genetic analysis. MARC-145 cell cultures were harvested at 4 dpi using a cell scraper, without freezing and thawing, to ensure high yield extraction of intact viral RNA. A 300-*µ*L mixture of detached cells and media was lysed with 700 *µ*L of TRI agent according to the manufacturers' instructions. Host DNA in the extracted nucleic acid sample was removed using DNase included in the Direct-zol RNA Miniprep kit.

RNA quantification from MARC-145 cell cultures infected with SNUVR220803 was performed using the Quant-it™ RiboGreen RNA Assay Kit (Invitrogen, Carlsbad, CA). These purified RNA samples were sent to Macrogen Inc. (Seoul, Korea) for next-generation sequencing (NGS). An RNA sample with an RNA integrity number (RIN) above 7.0 was selected using TapeStation RNA ScreenTape Analysis (Agilent Technologies, Seoul, Korea). A total of 0.5 *µ*g of the selected sample was prepared for the library using the TruSeq Stranded Total RNA Library Prep Kit (Illumina, San Diego, CA), which includes an rRNA removal process. cDNA synthesis was carried out with SuperScript Ⅱ reverse transcriptase (Invitrogen, Carlsbad, CA) and random primers, followed by second-strand cDNA synthesis with DNA polymerase Ⅰ, RNase H, and deoxyuridine triphosphate (dUTP). The protruding ends of the cDNA were converted to 5′-phosphorylated and blunt-ended DNA by an end-repair process using T4 DNA polymerase and T4 polynucleotide kinase. Terminal transferase was applied to add an “A” base to the 3′ ends, followed by adaptor ligation. The library was quantified and qualified using KAPA Library Quantification Kit (Kapa Biosystems, Wilmington, MA) and TapeStation D1000 ScreenTape (Agilent Technologies). The indexed library underwent paired-end sequencing analysis in a NovaSeq 6000 system (Illumina, San Diego, CA).

Sequence read data were examined for quality with FastQC (Version 0.12.0), and reads paired with the host genome were separated using Samtools (version 1.19). Filtered cDNA reads were mapped to the reference genomes of representative strains from Lineage 1 to 5, including NADC30 (GenBank no. MH500776) and VR-2332 (GenBank no. AY150564), and subjected to de novo assembly using Geneious Prime (Version 2023.2).

### 2.5. Genetic and Phylogenetic Analysis

The assembled genomic sequence of the SNUVR220803 was aligned using the MUltiple Sequence Comparison by Log-Expectation (MUSCLE) algorithm with whole-genome sequences of PRRSV strains available on GenBank, including the most similar sequences in the National Center for Biotechnology Information (NCBI) Basic Local Alignment Search Tool (BLAST) search results. Phylogenetic trees based on ORF5 sequences and whole-genome sequences were generated using RAxML (Version 8.2.10) and set to perform 1,000 bootstrap replicates using the GTRGAMMA model. A total of 100 whole-genome sequences were selected, comprising Korean PRRSV-2 strains and representative strains from each lineage currently available, including the genomic sequence of SNUVR220803. The results were visualized with FigTree (Version 1.4.4).

The restriction fragment length polymorphism (RFLP) pattern of the ORF5 gene was analyzed by treating the ORF5 PCR product, which was produced using primers from a preceding article [12], with Mlu Ⅰ, Hinc Ⅱ, and Sac Ⅱ. The classification of RFLP patterns was verified through in silico identification of restriction enzyme recognition sites.

### 2.6. Nsp2 INDEL Pattern Analysis

**Figure 1:**
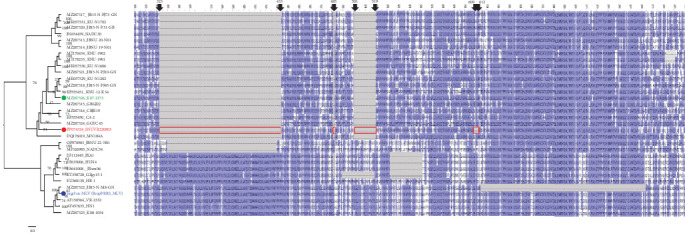
INDEL pattern of Nsp2 amino acid sequence of SNUVR220803. On the left side of the figure, the phylogenetic tree of Nsp2 amino acid sequences from 32 strains is shown, consisting of Korean PRRSV Type 2 strains and other representative strains from Lineages 1, 5, and 8. The major parental strain of the recombination event of the SNUVR220803 strain is shown with the green circle, and the blue circle represents the minor parental strain. Amino acid sequences from the right side show 300^th^ to 800^th^ amino acids in Nsp2 of each strain at the phylogenetic tree, which is a part of the HV region in the Nsp2 sequence. Deleted amino acids of the SNUVR220803 strain based on the VR-2332 strain are shown with red boxes, and the location of the deletion in the Nsp2 amino acid sequence is shown above the sequences with arrows and their numbers.

A total of 32 Nsp2 amino acid sequences from Korean PRRSV-2 strains and other strains from Lineage 1, 5, and 8 were compiled and aligned using the MUSCLE algorithm. A phylogenetic tree was constructed using the RAxML PROTGAMMAJTT model, incorporating 1,000 bootstrap replicates. Nsp2 deletion patterns were compared against the Nsp2 protein sequence of VR-2332, visualized in Jalview (Version 2.11.3.0), and colored using the BLOSUM62 matrix (Figure 1).

### 2.7. Recombination Analysis

A total of 334 whole-genome sequences of PRRSV-2 strains were selected for recombination analysis. This set included Korean PRRSV-2 strains, available modified live vaccine strains, representative strains of various lineages, and sublineage strains from Lineage 1 (*Supplementary 1*). Potential recombinant events were identified using RDP4 (Version 4.101), which conducted a comprehensive recombination scan employing seven methods (RDP, GENECONV, Bootscan, MaxChi, Chimaera, Sister Scanning, and 3Seq). A similarity plot was generated with Simplot (Version 3.5.1), comparing reference sequences of potential parent strains against SNUVR220803. The most suitable parent strains were determined based on the epidemiologic status of Korean PRRSV, with all qualifying strains undergoing these tests.

### 2.8. Experimental Design of the Animal Experiment

A total of 24 pigs at 3 weeks of age were purchased from a farm certified as free of PRRSV. The pigs were vaccine-naïve for both PRRSV and PCV2. Blood and nasal swabs were pulled from individual pigs and examined for PRRSV, PCV2, and *M. hyopneumoniae* prior to selection. Samples were pathogen negative for all three, as evaluated by real-time PCR results and enzyme-linked immunosorbent assays (ELISA). Purchased pigs (12 males and 12 females, 24 total) weighed between 5.43 and 6.22 kg and were bought from four different sows, six piglets per sow.

Pigs were randomly allocated into four different cages, each containing six pigs per cage based on ear notch labeling. Cages were identical in size, temperature, and humidity that were controlled by separate energy recovery ventilators. The pigs were provided with ad libitum access to feed and water. The infected groups contained 18 pigs, 12 were directly challenged with SNUVR220803, while the other six (sentinels) were used for indirect transmission. The 12 infected pigs were inoculated intranasally with 3 mL of filtered cell culture supernatant of SNUVR220803 (3rd passage in MARC-145 cells, 1.2 × 10^5.0^ tissue culture infective dose 50% (TCID_50_/mL)). The six noninoculated sentinel pigs resided in the same cages as the inoculated group. The negative control group was comprised of the remaining six pigs, each of which received an intranasal inoculation with 3 mL of sterile cell culture supernatant from MARC-145 cells. The study group size was determined based on the number of SNUVR220803-inoculated pigs that were expected to show clinical signs and the availability of statistical analysis.

Pigs were observed for 14 dpi, and scores of respiratory clinical signs, activity, body condition, and rectal temperature of each pig were recorded every day. Respiratory clinical sign scores were assigned within a range of 0–6 [13], activity scores between 1 and 5 [14], and body condition scores within 1–5 [15] following a scale developed from preceding research. Briefly, respiratory clinical signs ranged from 0 to 6: 0 indicated normal, 1 mild dyspnea and/or tachypnea when restrained, 2 mild dyspnea and/or tachypnea at rest, 3 moderate dyspnea and/or tachypnea when restrained, 4 moderate dyspnea and/or tachypnea at rest, 5 severe dyspnea and/or tachypnea when restrained, and 6 severe dyspnea and/or tachypnea at rest. Pigs were restrained daily by the handler holding them under his arm while taking their rectal temperature for 30–60 s. Activity levels ranged from 1 to 5: 1 indicated calm with little movement, 2 slow-paced movement, 3 continuous rapid movement, 4 continuous rapid movement with vocalization, and 5 continuous movement with vocalization and escape attempts. Body condition was scored from 1 to 5: 1 indicated prominent pelvic bones, narrow loin, hollow flank, visible vertebrae, and prominent ribs; 2 indicated obvious pelvic bones, narrow loin, somewhat narrow flank, visible vertebrae, and less apparent ribs; 3 indicated covered pelvic bones, covered vertebrae, and palpable ribs; 4 indicated undetectable pelvic bones, vertebrae, and ribs by palpation; and 5 indicated the same as score 4 with the midline slightly hollow between rolls of fat.

At 0, 4, 7, 10, and 14 dpi, body weight was measured, and the average daily weight gain (ADWG) was calculated as the difference between each measuring points divided by the difference of days between each pair of the points. At the same time points, blood samples were collected for quantification of PRRSV viremia and cytokine including tumor necrosis factor-*α* (TNF-*α*, Porcine TNF-alpha Quantikine ELISA kit, R&D systems inc., Minneapolis, MN), interleukin 6 and 10 (IL-6 and 10, Porcine Quantikine ELISA kit, R&D systems inc., Minneapolis, MN), and interferon-*α* (IFN-*α*, Porcine IFN-alpha ELISA kit, Invitrogen, Carlsbad, CA). Pigs were euthanized by electrocution on 14 dpi, and tissue samples of the cerebrum, cerebellum, nasal turbinate, mandibular gland, tonsil, thyroid gland, thymus, laryngeal cartilage, esophagus, lung, heart, liver, stomach, ileum, colon, spleen, mesenteric lymph node, kidney, inguinal lymph node, and dermis were collected. PRRSV cDNA genomic copies in the tissues were quantified with the described method in Section 2.10, and their paraffin blocks were made for hematoxylin and eosin staining (H&E staining) and immunohistochemistry. Researchers who recorded scores and performed necropsy did not know the allocated group of each cage apart from one who distributed the pigs into groups and treated pigs with the virus inoculation. The data collected from pigs that deceased during the experiment were included in the analysis.

A humane endpoint was established for the pigs in this experiment, where an experienced veterinary specialist in laboratory animals would consider euthanasia if their cumulative score reached or exceeded 3, based on the following assessment system: Weight change—0 (normal), 1 (body weight loss less than 10%), 2 (weight loss between 10% and 15%), and 3 (weight loss more than 20%); appearance change—0 (normal), 1 (loss of normal behavior), 2 (roughened hair and nasal and eye discharge), and 3 (very rough hair and abnormal posture); and behavioral change—0 (normal), 1 (subtle changes such as limping or interest in the injection site), 2 (abnormalities such as reduced behavior or activity loss), and 3 (vocalization, self-harm, severe anxiety, or motionlessness). Despite these criteria, no pigs were euthanized during the experiment, as those that reached or exceeded a score of 3 prematurely succumbed to the effects of the etiological agent, PRRSV SNUVR220803, before a decision on euthanasia could be made. Consequently, no other anesthetics or chemical agents were used throughout the experiment. Experimental methods were approved by the Seoul National University Institutional Animal Care and Use and Ethics Committee (IACUC, SNU-221220-1-1).

### 2.9. RNA and cDNA Preparation

RNA was extracted with RNAiso Plus (Takara Bio, Otsu, Japan) from clinical samples. Each tissue sample, weighing 0.1 g, was thoroughly grounded under liquid nitrogen in 300 *µ*L of sterile RNase-free PBS. Equal volumes with the PBS (300 *µ*L) of plasma or homogenized tissue samples were vortexed with 700 *µ*L of RNAiso Plus, following the manufacturers' instructions. Recombinant RNase Inhibitor (Takara Bio, Otsu, Japan) was added to all RNA samples. The number of freeze–thaw cycles outside −80°C deep freezer was limited to two. cDNA was synthesized using TOPscript™ Reverse Transcriptase (Enzynomics, Daejeon, South Korea) and random hexamer primers, followed by real-time PCR to quantify PRRSV cDNA.

### 2.10. Detection and Quantification of PRRSV cDNA

Before identification of the whole genome of SNUVR220803, for PRRSV detection, SYBR green real-time PCR assay targeted the ORF7 gene of SNUVR220803 (GenBank accession no. PP074324) using the primers SNUNA-22F (5′-CAAATAACAACGGCAAGCAG-3′) and SNUNA-22R (5′-AAATGGGGCTTCTCCGG-3′). Post-NGS, the TaqMan™ assay was applied to quantify cDNA samples from in vivo experiments, following the methodology of Wei et al. [16].

For viral RNA copy number quantification in in vivo samples, a recombinant plasmid based on the ORF7 sequence of SNUVR220803 (372 bp) was cloned into a pBHA vector (Bioneer, Daejeon, Korea). The concentration of the cloned plasmid was determined using a microplate spectrophotometer (Bio Tek Epoch, Winooski, VT). The DNA copy number of plasmid solution was calculated based on its molecular weight. A linear plot between the quantification cycle (Cq) value from diluted plasmid in TaqMan™ real-time PCR and the estimated DNA copy number on a logarithmic scale was created. A standard curve from this linear regression was used to calculate the genomic copy number of the SNUVR220803 strain from the Cq values of cDNA samples.

### 2.11. Immunohistochemistry

Tissues obtained from euthanized pigs were fixed in 10% formalin buffer for 1 day and embedded in paraffin. Sections of paraffin blocks, set at a 3-*µ*m thickness using a microtome, were attached to slides. The slides underwent a hydration process and were permeabilized with 0.05% Triton X-100 in Tris-buffered saline containing Tween 20 (0.1% TBST; 100 mM, pH 7.6). For antigen retrieval, 0.5% proteinase K (Invitrogen, Carlsbad, CA) in TBST was applied in a 37°C incubator for 15 min. Slides were then incubated overnight at 4°C with SR30 monoclonal antibody, diluted 1 in 2,000 in TBST. Subsequently, Goat anti-Mouse IgG (H + L) secondary antibody conjugated with alkaline phosphatase (Thermo Fisher Scientific, Waltham, MA), diluted in 1 in 4,000 in TBST, was applied to the slides for 2 hr at RT. Staining was performed using the Vector® Red AP Substrate Kit (Vector Laboratories, Newark, CA).

### 2.12. Morphometric Analysis

For the evaluation of the extent of pulmonary lesions, lung morphometric analysis was conducted. Each lung lobe was scored based on its estimated contribution to the total lung volume, as previously described by Halbur et al. [17]. The scores of affected lung lobes were summed to quantify the overall lung pathology for each experimental pig.

Microscopic evaluation of pulmonary lesions was performed through morphometric analysis. Lung tissue sections were reviewed blindly to assess the intensity of interstitial pneumonia, using an established scoring system [17]. The scoring was as follows: 0 indicated no lesions; 1 denoted mild interstitial pneumonia; 2 corresponded to moderate, multifocal interstitial pneumonia; 3 signified moderate, diffuse interstitial pneumonia; and a score of 4 indicated severe pneumonia.

### 2.13. Statistical Analysis

To assess the distribution of all numeric variables, the Shapiro–Wilk test was performed to determine data normality. A Shapiro–Wilk result below 0.05 indicated a deviation from normal distribution, leading to the use of a nonparametric test. Consequently, the Kruskal–Wallis test was utilized for overall comparisons, followed by pairwise Mann–Whitney tests, which were adjusted by using the Holm–Bonferroni method.

For variables that conformed to a parametric model, a one-way analysis of variance (ANOVA) with Tukey's post hoc adjustment was applied, except for the analysis of ADWG. The ADWG was assessed using analysis of covariance (ANCOVA), accounting for initial body weight as a covariate to detect the differences among the inoculated, sentinel, and control groups. All statistical analyses were conducted using the SPSS version 26.0 software (SPSS Inc., Chicago, IL).

## 3. Results

### 3.1. In Vitro Identification of Virus

The virus was first successfully isolated in MARC-145 cells and identified in the cytoplasm of PAM cells with IFA methodology for the detection of the PRRSV antigen. The virus was propagated in MARC-145 cells, and a plaque assay was performed. The titer of the SNUVR220803 strain in the supernatant measured over 10^5^ TCID_50_/mL at 4 dpi, which was similar to the other LKC strain (SNUVR240314). In contrast, the titer of RespPRRS_MLV reached this level at 2 dpi. The results of the IFA, plaque assay, and virus titration are visualized in *Supplementary figure 2*.

### 3.2. Phylogenetic Analysis

**Figure 2:**
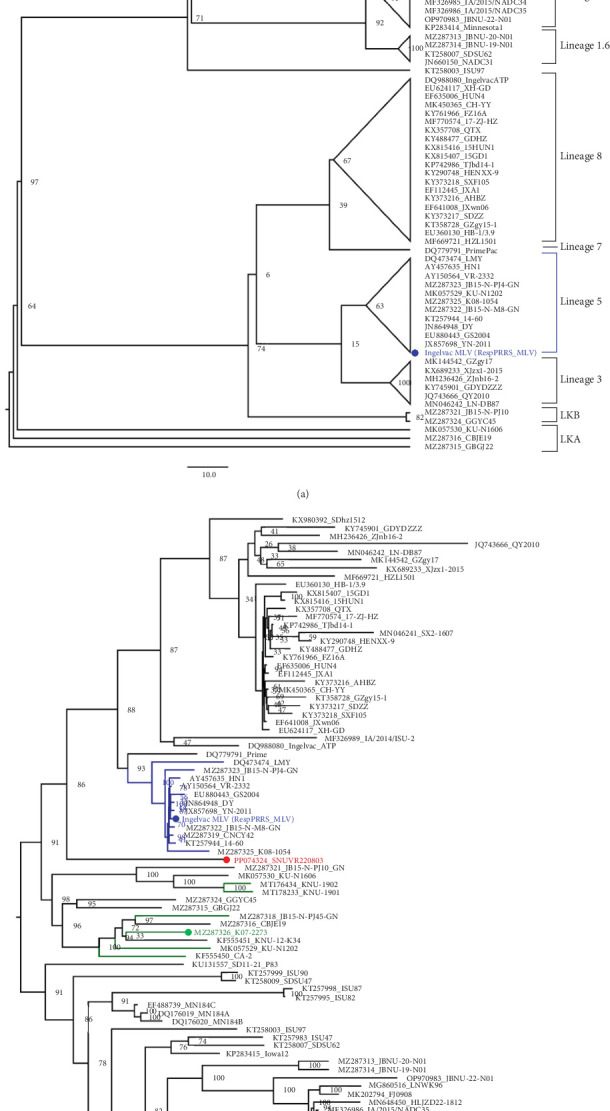
Phylogenetic trees based on ORF5 genes and whole genomes of the PRRSV-2 strains. In (a), 100 strains of PRRSV-2 are classified with their lineages in cartoon style based on ORF5 genes, and their location follows an increasing order. In (b), the phylogenetic tree is generated with whole genomes of the same strains as those of (a) and shown as a midpoint root tree. In terms of the recombination event, the major parental strain is marked with a green circle, and the minor parental strain is shown with a blue circle. Both trees are generated with 1,000 bootstrap tests, and the bootstrap numbers are noted at each of the diverging points of the phylogenetic trees.

Phylogenic trees based on ORF5 sequences were visualized with nodes in increasing order, and the classification is shown with lineages of PRRSV-2 (Figure 2) [4]. SNUVR220803 was solely clustered with other six LKC strains, which classifies it as LKC when referring to the definition of lineage Kor strains [5]. However, the phylogenetic tree based on whole-genome sequences indicates that SNUVR220803 shares a same root with Lineage 5 strains which differs it from that of the other LKC strains. Furthermore, a whole genome of SNUVR220803 shares only 84.47%–86.36% of its base pairs with other LKC strains, which makes it more divergent from these strains than the difference of pairs between each of the six LKC strains. Similarities of other gene segments of SNUVR220803 with PRRSV-2 strains are detailed in Table 1.

### 3.3. Nsp2 INDEL Pattern and ORF5 RFLP Analysis

Seven LKC PRRSV whole genomes were released and available for use up until this point in GenBank. SNUVR220803 is unique to other strains, with 135-aa deletions including additional four amino acid deletions at position 609–612 in the VR-2332 Nsp2 amino acid sequence, which consists of the “111+1+19+4” deletion pattern in the hypervariable (HV) region (315–855 aa) of the Nsp2 amino acid sequence. Nsp2 amino acid sequences of the other LKC strains have 131-aa discontinuous deletions including 111-aa deletion at position 323–433 in VR-2332, 1-aa deletion at position 485, and 19-aa deletions at position 501–519 which are the same as the NADC30-like strains and Korean lineages A and B (Figure 1) [7]. The Nsp2 sequence of SNUVR220803 shares 79.83% identity with that of GGYC45 (GenBank no. MZ287324).

In the Nsp2 amino acid sequence, SNUVR220803 exhibits 81.31%–85.16% similarity with other LKC PRRSV strains and 73.18% similarity to VR-2332, the prototype of classical PRRSV-2. Among the six reported LKC PRRSV strains, excluding SNUVR220803, the similarity of Nsp2 amino acid sequences ranges from 84.41% to 95.93%.

The ORF5 RFLP of SNUVR220803 was revealed as 1-2-4 which is same as HB17A, JB15-N-PJ73-GN, and HENAN-XINX (GenBank no. MG844181, KF611905, and MZ287317 for each) strains belonging to NADC30-like strains [18, 19, 20].

### 3.4. Recombination

**Figure 3:**
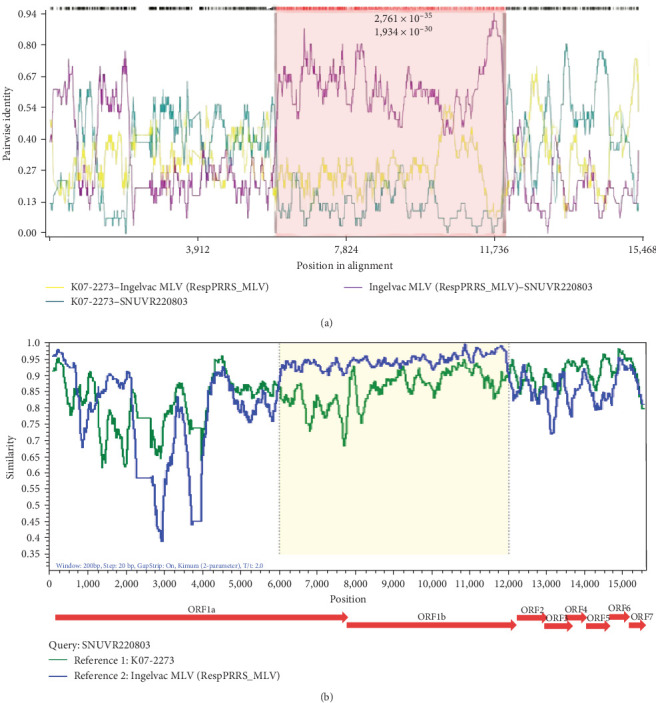
Recombination analysis of the SNUVR220803 strain between K07-2273 strain and Ingelvac MLV strain. (a) A recombination event ranging from 5,764 to 11,804 nt of SNUVR220803 based on the nucleotide sequence of VR-2332 is detected from the RDP4 software. Each color of the lines represents pairwise identity between the genomic sequence of SNUVR220803 and the parental strains. Expected recombination site is colored with a red box. (b) Similarity plot from the Simplot software is visualized, which represents the similarity between genomic sequences of the query strain (SNUVR220803) with the reference strains as the parental strains. The recombination site is also marked with a yellow color, and the arrows behind the similarity plot show the location of protein-coding sequences based on those of VR-2332.

Within RDP4 analysis, the whole genome of SNUVR220803 was detected as the recombinant strain whose major parent is K07-2273 (GenBank no. MZ287326), one of the LKC strains, and the minor parent is RespPRRS_MLV (Ingelvac MLV, GenBank no. AF066183), a strain of modified live vaccine belonging to Lineage 5 (Figure 3). Results of all seven incorporated methods in RDP4 (RDP, GENECONV, Bootscan, MaxChi, Chimaera, Sister Scanning, and 3Seq) were positive about this recombinant event.

Simplot analysis with SNUVR220803 as a query and K07-2273 and RespPRRS_MLV as parental strains suggested that the crossovers took place between the ORF1a and ORF1b regions. The breakpoints of this major recombination were found in 5,764–11,804 nt regarding the genomic sequence of VR-2332 which are in range from Nsp4 to Nsp12. Other possible recombination events of SNUVR220803 from parental strains K07-2203 and RespPRRS_MLV were detected in the 5′ UTR to Nsp1 region (39–508 nt) and in Nsp1 to Nsp2 (1,254–2,103 nt), which are described in Table 2.

### 3.5. Clinical Observation

**Figure 4:**
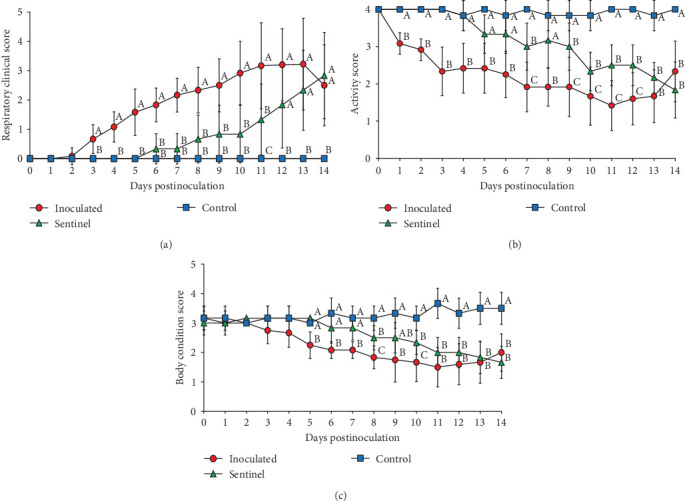
Clinical scores: (a) respiratory clinical score, (b) activity score, and (c) body score. The significantly different groups (*P* < 0.05) are marked with different superscripts, including (A), (B), and (C).

**Figure 5:**
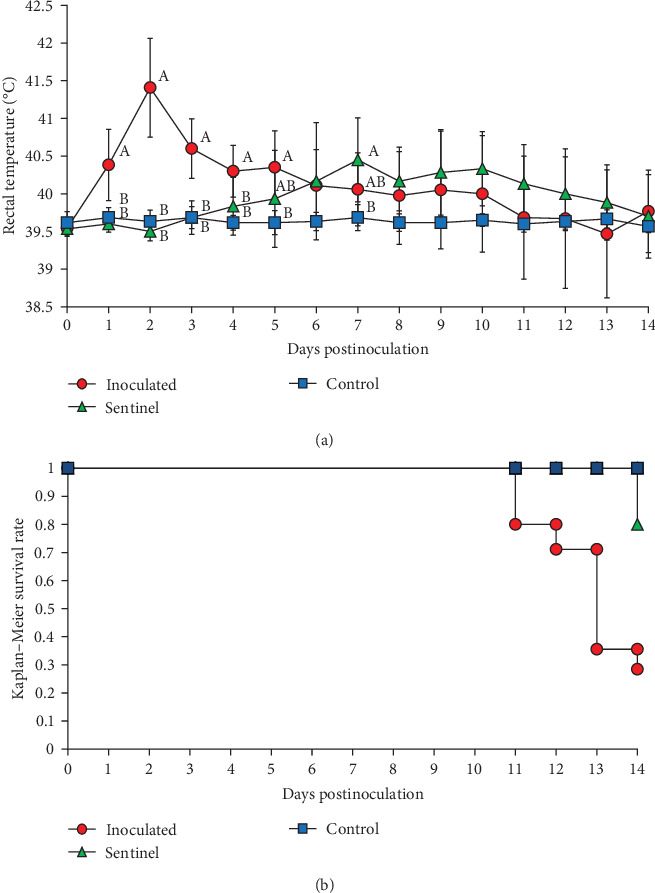
Rectal temperature and survival rate curve. (a) Rectal temperature data are shown, and groups significantly different (*P* < 0.05) are marked with alphabets, including (A), (B), and (C). (b) Kaplan–Meier survival rates.

The control group which received a mock inoculation did not show respiratory clinical signs, and the activity score and the body condition score were normal. The mean respiratory clinical sign score was significantly higher in the group inoculated with SNUVR220803 from 4 to 14 dpi. The pigs in the sentinel group showed higher clinical sign scores than the control group from 11 to 14 dpi. From 1 to 14 dpi, the group inoculated with SNUVR220803 indicated significantly lower activity score and body condition scores than the control group. The sentinel group showed a lower activity score than the control group from 7 dpi. At 8 dpi and 10–14 dpi, the pigs in the sentinel group had lower body condition scores than those in the control group (Figure 4). The pigs inoculated with the SNUVR220803 strain died at 11 dpi (two pigs), 12 dpi (one pig), 13 dpi (three pigs), and 14 dpi (one pig) (Figure 5). A pig in the sentinel group was found deceased at 14 dpi.

The rectal temperature measured significantly higher in the inoculated group than in the control group at 1–5 dpi, with the sentinel group having the highest rectal temperature at 7 dpi (Figure 5). The peak rectal temperature occurred at 2 dpi as the mean of the inoculated group reached 41.41°C and then decreased markedly at 3 dpi. The fluctuation of rectal temperature variated between individual pigs in the inoculated and sentinel groups from 10 to 14 dpi, and the temperature of deceased pigs plunged down between 38.3 and 39.3. The rectal temperature of the control group remained normal and stable throughout the experiment.

Most of the inoculated pigs (11/12 pigs) lost weight during at least one time point of the measurement. The mean body weight of the inoculated group was lower at 4–14 dpi than at 0 dpi (5.83 kg) (Table 3). At 14 dpi, the inoculated group measured the lowest in body weight, followed by the sentinel group, and then the control group. Inoculated pigs that died prior to the end of the experiment measured as significantly lower in body weight at 7–10 dpi than pigs that survived from the same group. The ADWG of the inoculated group was significantly lower than that of the sentinel and control group in the time intervals between 0–4 dpi, 4–7 dpi, and 0–14 dpi. The sentinel group had a lower ADWG than the control group between 0–4 dpi and 0–14 dpi.

**Figure 6:**
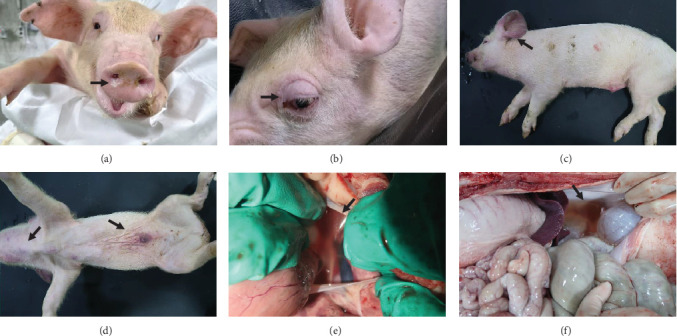
Gross lesions found in pigs infected with SNUVR220803. Gross lesions of the pigs infected with SNUVR220803 are arranged. Each arrow of (a–f) indicates the following lesions found in live and necropsied pigs: (a) white nasal discharge, (b) eyelid edema, (c) and (d) cyanosis in extremities, (e) pericardium effusion, and (f) peritoneal effusion.

Pigs in the inoculated group experienced emaciation, roughened hair coat, and erythematous rash at 7 dpi. Eyelid edema and cyanosis on the belly and ear skin were observed at 11 dpi. Pigs from the SNUVR220803 group that died during the experiment exhibited white nasal discharge, shivering, inappetence, tachypnea, and labored breathing (Figure 6).

### 3.6. PRRSV Quantification with Real-Time PCR

**Figure 7:**
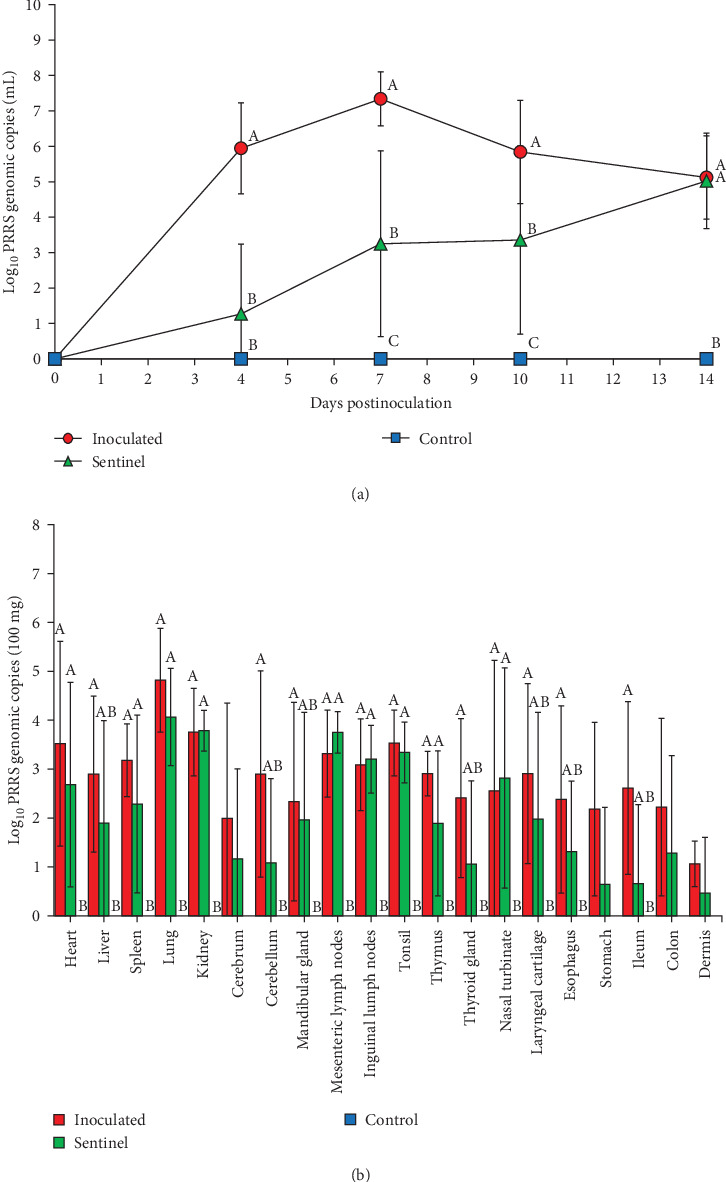
Quantification of PRRSV genomic copies in viremia and the tissue extracts. (a) The graph of logarithmic PRRSV genomic copy values in viremia of experimental pigs. Different superscripts including (A), (B), and (C) indicate statistically different groups (*P* < 0.05). (b) PRRSV genomic copies in 100 mg of each tissue samples.

PRRSV RNA was not detected in the plasma and tissue samples of the negative control group during the experiment. The peak of PRRSV viremia occurred at 7 dpi in the SNUVR220803-inoculated group as measured by 10^7.3^ genomic PRRSV copies, where viremia persisted until the experiment ended at 14 dpi (Figure 7). Deceased pigs in the inoculated group had significantly greater PRRSV genomic copies in the plasma than pigs. The inoculated group had significantly higher viremia than the sentinel and control groups at 4–10 dpi. The sentinel group measured higher in plasma genomic copies than the control group at 7–10 dpi. At 14 dpi, viremia of both the inoculated and sentinel groups was significantly higher than the control group.

All tested tissue samples from the 20 organs listed in Section 2.7 of the SNUVR220803 group resulted positive in at least five pigs (Figure 7). The mean value of PRRSV genomic copies in 100 mg of lung sample was the highest at 10^4.8^ copies. With an exception to the cerebrum, stomach, colon, and dermis samples, the inoculated group was statistically higher in the amount of measured PRRSV cDNA than the control group. The sentinel group measured significantly higher in PRRSV genomic copies from the heart, spleen, lung, kidney, lymph nodes, tonsil, thymus, and nasal turbinate than the control group. All samples that yielded a positive result for PRRSV cDNA through quantitative PCR testing were fixed for additional testing with immunohistochemistry. Of the samples tested with immunohistochemistry, only certain organs (the lung, lymph nodes, thymus, heart, kidney, and sulcus of cerebrum) were indicated as positive for PRRSV antigen.

### 3.7. Plasma Cytokine

Pigs inoculated with the SNUVR220803 strain exhibited significantly higher plasma TNF-*α* levels compared to both the sentinel and control groups at 4 dpi. The level of TNF-*α* further increased within the inoculated group, as having the highest TNF-*α* levels, followed by the sentinel group and then the control group at 7 and 10 dpi. By 14 dpi, TNF-*α* levels were alleviated in the inoculated group, and both inoculated and sentinel groups displayed statistically higher TNF-*α* levels compared to the control group.

The inoculated group exhibited the highest IFN-*α* levels at 4 dpi, followed by the sentinel and then the control group. By 7 and 10 dpi, IFN-*α* in the inoculated group had mostly returned to low levels, and both the inoculated and sentinel groups were statistically higher in IFN-*α* levels compared to the control group. At 14 dpi, the sentinel group had the highest levels of IFN-*α*, followed by the inoculated and then the control group.

Significant differences in the amount of measured IL-6 were not observed among the groups throughout the duration of the experiment, although the mean IL-6 value did increase in the inoculated group until 10 dpi.

**Figure 8:**
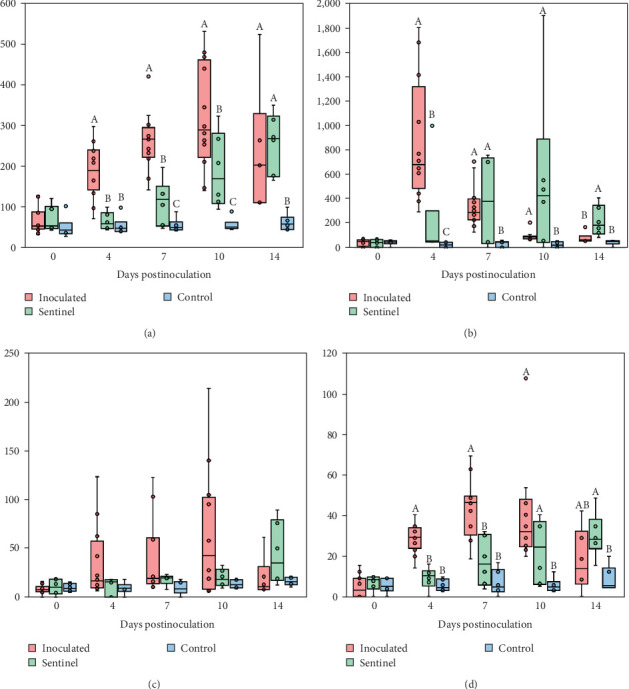
Cytokine levels in the plasma. The data of (a) TNF-*α*, (b) IFN- *α*, (c) IL-6, and (d) IL-10 are shown with box graphs. Data out of range are shown as dots separate from the box graphs. Different superscripts including (A), (B), and (C) indicate statistically different groups (*P* < 0.05).

The inoculated group IL-10 levels measured higher than both the sentinel and control groups at 4 and 7 dpi. By 10 dpi, both the inoculated and sentinel groups had higher IL-10 levels compared to the control group despite a small decrease in the inoculated group IL-10. At 14 dpi, the sentinel group still surpassed the control group in IL-10 levels, while the inoculated group did not (Figure 8).

### 3.8. Pathology

Specific gross or histopathological lesions were not found in the negative control group pig tissues. Samples from pigs that died in the SNUVR220803 group prior to the end of the experiment (7/12) were examined and diagnosed with peritoneal and pericardial serous effusion in gross lesions (Figure 6). Additional fibrosis and pleuropneumonia were not found, and pathogens such as *Haemophilus parasuis*, *M. hyopneumoniae*, and *Mycoplasma hyorhinis* were not detected in the microorganism culture test or through real-time quantitative PCR of lung samples.

**Figure 9:**
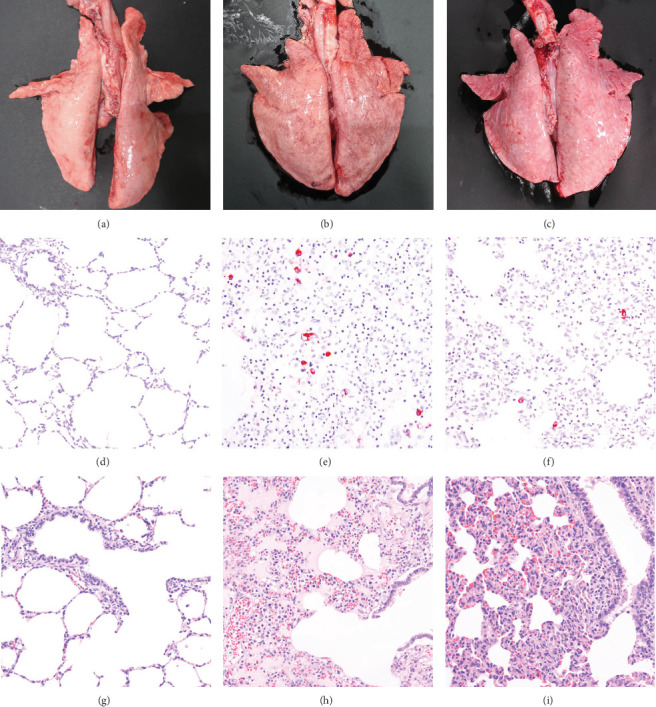
Lung lesions. (a–c) Show macroscopic lung lesions of the pigs in the control group and the inoculated group: control group (a), pigs in the inoculated group that deceased at 11 dpi (b), and at 14 dpi (c). (d–f) Show immunohistochemistry results in the lungs of the pigs in each experimental group: control group (d), pigs in the inoculated group that deceased at 11 dpi (e), and at 14 dpi (f). (g–i) Show HE staining of microscopic lung lesions of the pigs in each experimental group: control group (g), pigs in the inoculated group that deceased at 11 dpi (h), and at 14 dpi (i).

In pigs that died at 11 dpi in the inoculated group, histopathological lesions detected in the prepared HE-stained lung tissue slides confirmed pulmonary edema in the congested alveolar septa. Severe diffuse interstitial pneumonia with mononuclear cell infiltrates was observed in the lungs of pigs in the inoculated group at 14 dpi (Figure 9). The pigs in the inoculated and sentinel groups had statistically higher lung gross lesion scores than those in the control group. The inoculated group had the highest lung microscopic lesion scores, and the sentinel group had the second highest scores, followed by the control group (Table 3).

### 3.9. Immunohistochemistry

**Figure 10:**
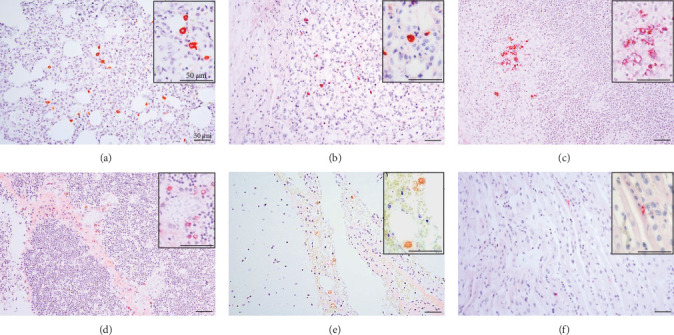
Immunohistochemistry about PRRSV antigen. The positive signals in immunohistochemistry of tissues from pigs infected with SNUVR220803 are arranged. The organs include (a) the lung, (b) the kidney, (c) lymph node, (d) thymus, (e) blood vessels in the cerebral sulcus, and (f) the heart. SR30 anti-PRRSV antibody is used for immunohistochemistry with alkaline phosphatase staining (red). The figures are in 200x magnification, and the insets are included (×400).

PRRSV antigen-positive cells were detected in the lungs, lymph nodes, heart, kidney, and the blood vessels of the cerebral sulci samples from the inoculated pig groups (Figure 10). In lung tissues of all SNUVR220803-inoculated pigs, macrophages that were irregularly spread in the alveolar space as well as septa found in the lungs were color red as positive cells for PRRSV antigen. Lymphocytes in the submandibular, mesenteric, inguinal lymph nodes, and thymus were detected as positive cells for PRRSV in immunohistochemistry (IHC). Macrophages from the kidney and heart tissue were also positive for PRRSV antigen. PRRSV was not detected in parenchymal cells from the cerebrum or cerebellum through IHC, unlike the PRRSV cDNA from central nervous system tissue, although macrophages in the cerebral sulci were detected as positive for PRRSV in the inoculated pig groups.

## 4. Discussion

The SNUVR220803 strain of LKC PRRSV was classified separately as nonlineage, rather than within the phylogenetic tree of Lineages 1–9 that were generated using the maximum likelihood method based on the ORF5 sequence [5, 7, 21]. The SNUVR220803 strain was most closely linked to Lineage 5 based on the phylogenetic analysis of the whole genome; a category is completely different from the phylogenetic analysis of ORF5. Therefore, the classification of LKC can be confirmed by a phylogenetic analysis of ORF5 and Nsp2. The molecular markers of LKC are 131-aa discontinuous deletions in Nsp2, including a 111-aa deletion at position 322–432, a 1-aa deletion at position 483, and a 19-aa deletion at position 504–522 that correspond to the NADC30 complete sequence. The additional deletion of four amino acids in the Nsp2 protein sequence to that of 131 deleted amino acid strains (including NADC30-like strains, CA-2, and K07-2273) needs to be further analyzed for their contribution to the virulence of SNUVR220803. This is particularly true, as an existing report concluded that a 30-amino acid deletion in the Nsp2 of HP-PRRSV was irrelevant to PRRSV pathogenicity [22].

In this study, infection with the LKC PRRSV strain (SNUVR220803) led to persistent high fevers (above 40.3°C), pronounced interstitial pneumonia, and increased viremia in the treated piglets. This strain demonstrated significant pathogenicity among the piglets, with all infected animals exhibiting symptoms (100% morbidity) and a mortality rate of 58.3% (7/12 pigs). The results confirmed the SNUVR220803 strain of LKC PRRSV as a highly virulent strain of PRRSV, aligning with the severe outbreak at the farm of origin for the SNUVR220803 strain.

The SNUVR220803 strain of LKC PRRSV had higher pathogenicity than the CA-2 strain, which is also included in LKC PRRSV. SNUVR220803 infection caused 58.3% mortality rate in pigs, while no CA-2 infected pigs were discovered deceased by the conclusion of the study [10]. The mean rectal temperature in SNUVR220803-infected pigs was greater than 41°C at 2 dpi, while CA-2 infected pigs only developed a moderate fever (40–40.5°C). Average body weight decreased in SNUVR220803-infected pigs by 10 dpi, compared to CA-2-infected pigs which gained body weight during all 35 days of the experimental period. SNUVR220803-infected pigs developed severe macroscopic lung lesions, compared with the mild lung lesions from CA-2-inoculated pigs, while both sets of pigs developed microscopic lung lesions and severe interstitial pneumonia [23].

Recombination can be an important factor in explaining the pathogenicity differences between two strains. Recombination has previously proven to increase virulence as shown in the case of JL580, a Chinese NADC30-like PRRSV strain, which underwent recombination with other PRRSV strains, which significantly increased its pathogenicity in comparison to HNjz15, a different NADC30-like PRRSV strain from China that did not undergo recombination [24, 25]. Even though infection with the nonrecombinant CA-2 PRRSV strain resulted in limited pig pathogenicity, there is a potential risk that its recombination with other virus strains could result in the emergence of highly virulent strains like SNUVR220803. The notably high pathogenicity of SNUVR220803 is believed to stem from the extensive recombination of large gene fragments between close relatives of the major parental K07-2273 strain (GenBank no. MZ287326) and the minor parental RespPRRS_MLV strain (GenBank no. AF066183). Recombination of SNUVR220803 with the minor parental RespPRRS_MLV strain 5′ UTR may also be important as the 5′ UTR is indispensable for PRRSV infectivity [26]. The combination of RespPRRS_MLV 5′ UTR and a K07-2273 3′ UTR may lead to an increase in virulence. Most recombinant strains of PRRSV have increased pathogenicity, but on occasion, recombination has the opposite effect [27]. Hence, although the recombination event leading to the emergence of SNUVR220803 took place some time ago, it required further evolutionary steps to come up with the highly pathogenic phenotype.

The virulence of LKC PRRSV (SNUVR220803) has some commonalities in the pathologic characteristics of HP-PRRSV in China, which belongs to Lineage 8. Infection by either strain resulted in mean rectal temperature above 41°C in the early phase of infection, purulent nasal discharge, cyanosis in extremities, persistent high fever, decreasing body weight throughout the experiment, and a high mortality rate above 50% before 14 dpi. The Chinese HP-PRRSV strains JXwn06 and WUH2 were responsible for a 100% morbidity rate and a mortality rate ranging from 70% to 100% among the affected pigs. Conversely, another Chinese HP-PRRSV strain, HuN4, did not lead to fatalities in pigs that were experimentally infected [28]. In both cases of infection by either the SNUVR220803 strain or the various HP-PRRSV strains, viral antigens were detected within cells across multiple organs, including the kidney and lymph nodes [29]. However, there are apparent differences between the HP-PRRSV and SNUVR220803 strains in terms of virus distribution and pathogenesis in pigs. HP-PRRSV has tissue tropism in gastric glands and neurons [30], but the SNUVR220803 strain was not detected in digestive glands or the nervous system with IHC, with an exception to macrophages detected in the cerebral sulci. HP-PRRSV induced clinical signs which included neurological signs, hemorrhages in the kidney and lymph nodes, and liquefactive necrosis in the brain [31, 32], all of which were absent in SNUVR220803-infected pigs.

High plasma TNF-*α* in pigs infected with the SNUVR220803 strain can be related to the pathogenesis of the virus. This contradicted previous research by López-Fuertes et al. [33] which indicated that PRRSV could lead to a reduction of TNF-*α* in the supernatant of phorbol myristate acetate (PMA)-stimulated pulmonary alveolar macrophages. The SNUVR220803-inoculated group measured higher in TNF-*α* levels at 4, 7, 10, and 14 dpi than those from the control group. Despite the virus's TNF-*α* suppression mechanisms, this cytokine still plays a significant role in the pathogenesis of the infection, which can be associated with inflammatory response and cell apoptosis in the lungs [34]. It suggests that in the specific case of the SNUVR220803 strain, the host's immune response can overcome viral suppression mechanisms, resulting in an inflammatory response that has pathological consequences.

IFN-*α*, a Type-Ⅰ interferon, induces an antiviral response by promoting cytokines related to immunosuppression while inhibiting cellular translation machinery and degrading ssRNA in affected cells [35]. Plasma IFN-*α* levels surged early on at only 4 dpi in the inoculated group and were downregulated greatly from 7 dpi. Despite these high levels of IFN-*α* at 4 dpi, the SNUVR220803-inoculated group was not sufficiently protected from PRRSV. In the research of Amadori et al. [36], an early response of IFN-*α* caused by a virulent PRRSV strain can be related to poor prognosis in weaner pigs. The mechanism underlying the disease-promoting effects of Type-Ⅰ interferons includes tissue cell proliferation suppression, immunosuppression, and direct tissue damage by apoptosis induction [35].

A surge of IL-10, an anti-inflammatory cytokine that is promoted by PRRSV infection, was measured from pigs in the inoculated group starting late at 7 dpi and persisted until 14 dpi. PRRSV can activate IL-10 production through NF-*κ*B and p38 MAPK pathways in PAMs [37]. IL-10 can be another virulence marker in PRRSV-infected pigs in the case of a late response [36]. IL-10 can dampen the host's ability to deal with effective immune response by promoting the differentiation of Type Ⅰ regulatory cells [36]. The increase in IL-10 observed in the inoculated group, and at later stages in the sentinel group, could reflect a virus-induced shift toward an immunosuppressive state, potentially facilitating virus survival and spread.

Despite of the lack of statistical differences of IL-6 among groups, IL-6 may still play a critical role behind the scenes. IL-6 influences the acute phase of inflammation, and its role may extend to promoting virus survival and a potentially exacerbating clinical disease [38]. Understanding the full scope of IL-6's involvement in SNUVR220803 infections would require further analysis.

## 5. Conclusions

This report presents the first comprehensive examination, both clinical and experimental, of the extensive outbreak of PRRS in Korea and identification of a particularly virulent new strain, SNUVR220803, that causes severe disease in pigs. This SNUVR220803 strain stands out for its exceptionally high virulence and infectivity among PRRSV strains currently prevalent in the field and is anticipated to significantly contribute to future PRRS epidemics.

## Figures and Tables

**Table 1:** Genetic identity in percentage in each of the gene segments between the strain SNUVR220803 and representative strains of PRRSV-2 belonging to LKC, Lineage 1, 5, and 8.

Gene segment name	MZ287326 K07-2273	KF555450 CA-2	MT178233 KNU-1901	MT176434 KNU-1902	MZ287318 JB15-N-PJ45-GN	KF555451 KNU-12-K34	MZ287315 GBGJ22	MZ287324 GGYC45	MF326985 IA/2014/NADC34	KU131568 SD11-21_P100	JN654459 NADC30	DQ176019 MN184A	Ingelvac MLV (RespPRRS_MLV)	AY150564 VR-2332	EF641008 JXwn06
	LKC						LKA	LKB	Lineage 1.5	Lineage 1.7	Lineage 1.8	Lineage 1.9	Lineage 5		Lineage 8
	Percentage identity responding to SNUVR220803 (nucleotide sequence)														
WGS	86.36	86.46	84.47	84.57	85.11	85.78	87.14	86.25	82.62	84.11	84.85	85.34	85.37	85.96	82.10
5′UTR	NA	94.71	92.11	92.11	NA	92.11	92.06	NA	92.11	91.58	92.63	92.11	96.30	96.30	91.53
ORF1a	83.51	83.76	81.25	81.26	82.06	82.46	84.20	84.18	79.19	80.86	82.65	83.88	80.13	80.16	75.38
Nsp1	87.12	88.37	83.33	83.16	86.4	87.67	89.15	88.63	82.81	82.55	86.20	86.20	87.33	87.50	83.94
Nsp2	79.79	79.51	75.37	75.41	78.21	77.78	78.80	79.83	72.10	75.68	78.51	78.51	66.97	67.07	61.12
Nsp3	87.74	88.34	86.10	85.87	84.68	86.32	86.92	88.04	83.03	84.98	81.61	87.07	84.16	84.01	81.76
Nsp4	84.97	84.31	86.44	86.44	84.31	84.31	85.29	84.80	84.15	84.97	86.44	84.80	90.03	90.03	86.27
Nsp5	83.14	83.73	80.98	80.59	82.55	82.94	84.31	85.10	84.51	82.55	86.86	92.16	93.33	93.33	86.86
Nsp6	87.50	87.50	85.42	89.58	85.42	89.58	91.67	89.58	91.67	93.75	93.75	100.0	100.0	100.0	95.83
Nsp7	81.08	81.72	83.40	83.91	80.44	80.57	87.00	82.50	81.34	81.47	84.68	83.78	92.15	92.02	85.46
Nsp8	86.67	85.93	87.41	88.15	85.93	84.44	91.11	90.37	87.41	88.15	89.63	91.11	94.07	94.07	91.85
ORF1b	89.19	87.86	86.17	86.44	87.86	88.80	92.64	90.63	85.57	87.54	87.61	86.88	94.60	94.60	88.52
Nsp9	87.71	87.81	85.42	85.83	86.30	87.08	91.98	90.83	86.15	87.86	88.54	87.24	94.38	94.38	89.79
Nsp10	90.78	87.91	87.15	87.23	90.17	90.85	93.42	90.93	85.56	87.23	85.41	86.55	95.16	95.16	87.68
Nsp11	89.24	87.00	88.34	88.49	87.14	88.79	94.77	91.63	85.35	89.39	89.99	88.19	97.31	97.31	89.54
Nsp12	90.69	89.18	83.33	83.77	88.74	90.04	90.04	87.45	83.55	84.42	86.58	84.42	90.04	90.04	84.20
ORF2	89.49	88.98	86.12	85.99	86.90	87.16	87.29	85.47	85.86	85.08	83.14	84.95	87.8	88.07	85.34
ORF3	90.72	90.07	89.54	89.41	86.80	86.14	83.92	83.53	82.35	83.79	82.09	81.96	84.44	84.44	84.44
ORF4	94.04	91.99	93.11	92.74	91.81	91.99	85.47	85.85	87.52	85.47	87.71	85.29	86.22	86.22	87.71
ORF5	91.87	89.72	87.40	87.73	87.73	89.22	85.07	83.91	86.24	86.24	86.07	86.57	85.74	85.74	86.57
ORF6	94.10	92.57	92.00	91.81	92.57	92.57	87.24	88.38	87.24	91.05	87.24	89.33	89.71	89.90	89.71
ORF7	92.47	88.71	90.05	90.59	87.10	87.63	87.63	87.63	87.10	88.11	87.90	88.17	91.13	91.13	87.63
3′UTR	NA	86.21	87.93	87.07	85.34	87.07	85.34	78.45	85.34	79.31	87.07	87.93	86.21	84.48	76.07

**Table 2:** Recombination events in the whole genome of PRRS strain SNUVR220803 between strains K07-2273(LKC) and Ingelvac MLV (RespPRRS MLV, Lineage 5).

Query	Breakpoint corresponding to VR-2332 (nt)			Major parent	Minor parent	*P*-value							
	No.	Beginning	Ending			RDP	GENECONV	Bootscan	MaxChi	Chimaera	Sister Scanning	3Seq	
SNUVR220803	1	39	508	K07-2273	Ingelvac MLV (RespPRRS_MLV)	1.855 × 10^−11^	N/A	4.042 × 10^−12^	8.866 × 10^−7^	3.217 × 10^−5^	6.333 × 10^−6^	4.709 × 10^−10^	
						8.954 × 10^−24^	N/A	1.700 × 10^−23^	6.178 × 10^−12^	1.258 × 10^−15^	3.541 × 10^−12^	7.908 × 10^−12^	
	2	1,245	2,103										
	3	5,764	11,804										
						2.761 × 10^−35^	4.962 × 10^−7^	2.240 × 10^−34^	4.368 × 10^−29^	1.327 × 10^−26^	3.059 × 10^−14^	3.730 × 10^−14^	

**Table 3:** ADWG, bodyweight, lung gross pathology, and lung histopathological scores of the pigs inoculated with the strain SNUVR220803 inoculated, contact, and control pigs.

Measures	Days postinoculation	Inoculated group (number of pigs)	Sentinel group (number of pigs)	Control group (number of pigs)
Body weight (kg)	0	5.83 ± 0.26 (12)	5.73 ± 0.15 (6)	5.87 ± 0.14 (6)
	4	5.57 ± 0.21 (12)	6.37 ± 0.27 (6)	6.73 ± 0.19 (6)
	7	5.52 ± 0.22 (12)	6.83 ± 0.34 (6)	7.46 ± 0.27 (6)
	10	5.33 ± 0.48 (12)	7.3 ± 0.39 (6)	8.25 ± 0.35 (6)
	14	5.62 ± 0.64^c^ (12)	7.84 ± 0.83^b^ (6)	9.65 ± 0.63^a^ (6)

ADWG (g/pig/day)	0–4	−64 ± 50^c^ (12)	160 ± 34^b^ (6)	216 ± 16^a^ (6)
	4–7	−17 ± 67^b^ (12)	153 ± 79^a^ (6)	244 ± 34^a^ (6)
	7–10	−66 ± 113 (12)	157 ± 88 (6)	261 ± 63 (6)
	10–14	−4 ± 79 (12)	135 ± 165 (6)	352 ± 92 (6)
	0–14	−14 ± 56^c^ (12)	151 ± 65^b^ (6)	270 ± 36^a^ (6)

Lung gross lesion score	11	85 ± 0 (2)	—	—
	12	80 (1)	—	—
	13	66.7 ± 10.41 (3)	—	—
	14	60 ± 15.81^a^ (6)	52.5 ± 24.8^a^ (6)	0 ± 0^b^ (6)

Lung microscopic lesion score	11	3.1 ± 0.71 (2)	—	—
	12	2.6 (1)	—	—
	13	2.7 ± 1.01 (3)	—	—
	14	2.6 ± 0.74^a^ (6)	1.8 ± 0.8^b^ (6)	0.3 ± 0.4^c^ (6)

Standard deviation was calculated across the three groups to measure the variation, with distinct superscripts (a–c) employed to denote statistically significant differences (*P* < 0.05).

## Data Availability

Datasets used and/or analyzed during this study can be obtained from the corresponding author upon reasonable request.

## Supplementary Materials

Supplementary 1 A list of 333 strains of porcine reproductive and respiratory syndrome virus Type 2 (PRRSV-2) used in recombination analysis using RDP4, featuring Korean PRRSV-2 clades (LKA, LKB, and LKC), modified live vaccine strains, representative strains from nine lineages, and sublineage strains from Lineage 1 (Lineage 1.1, 1.2, 1.4, 1.5, 1.6, 1.7, 1.8, and 1.9).

Supplementary 2 Figure 1: immunofluorescence assay (IFA), plaque assay, and virus titer results for SNUVR220803. (a) Shows the IFA results for PAM cells that were either mock-inoculated or inoculated with SNUVR220803. (b) Demonstrates plaque formation in MARC-145 cells inoculated with SNUVR220803. (c) Compares the virus titers of SNUVR220803, another LKC SNUVR240314 strain, and RespPRRS_MLV.
