# Supplementary figures and images for: Genetic and Pathogenic Characteristics of an Emerging Highly Virulent Recombinant Lineage Korean Clade C PRRSV Strain
Source: Transbound Emerg Dis. 2024 Aug 12;2024:5785557. doi: 10.1155/2024/5785557 (PMC12016962; doi:10.1155/2024/5785557)

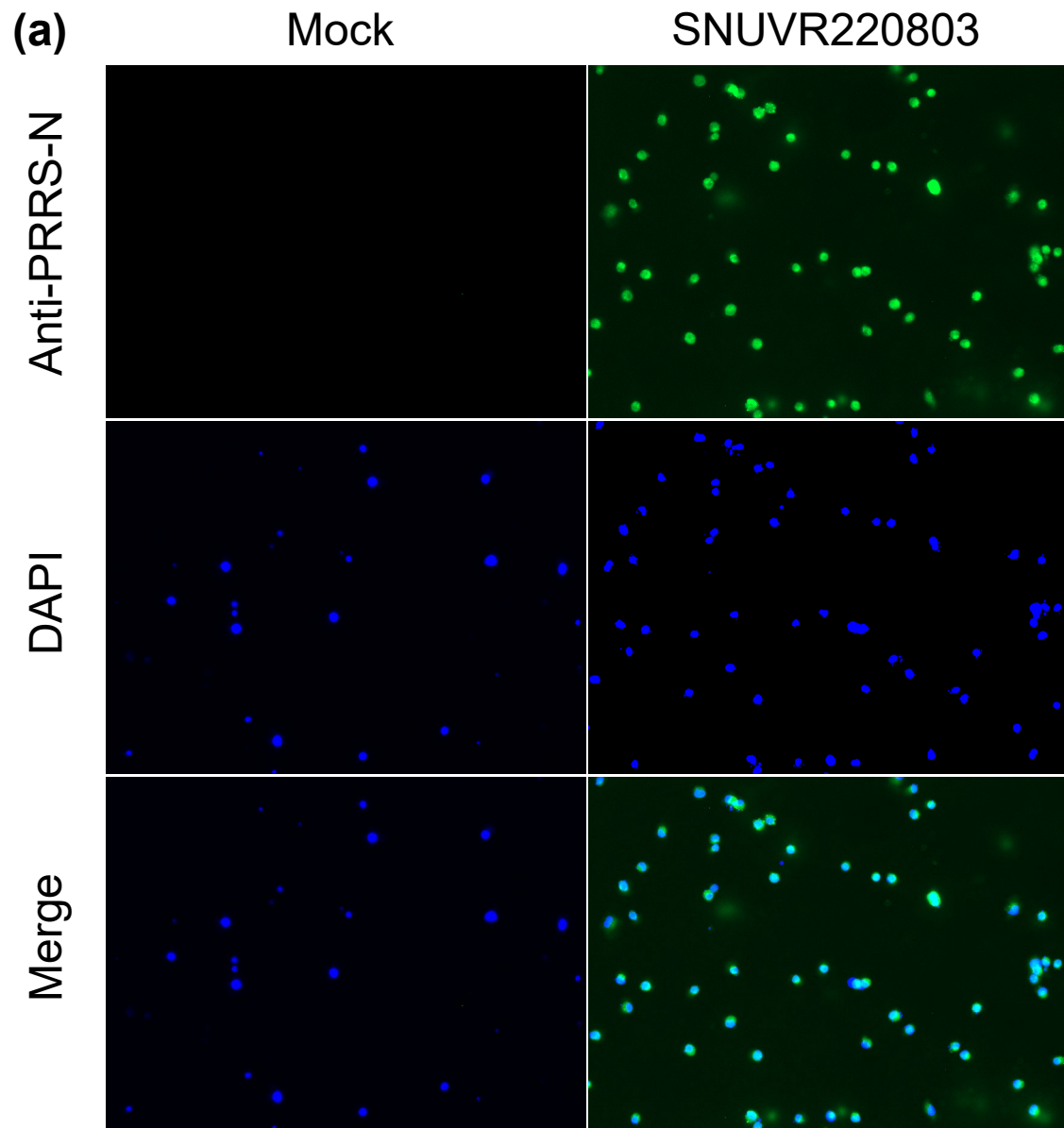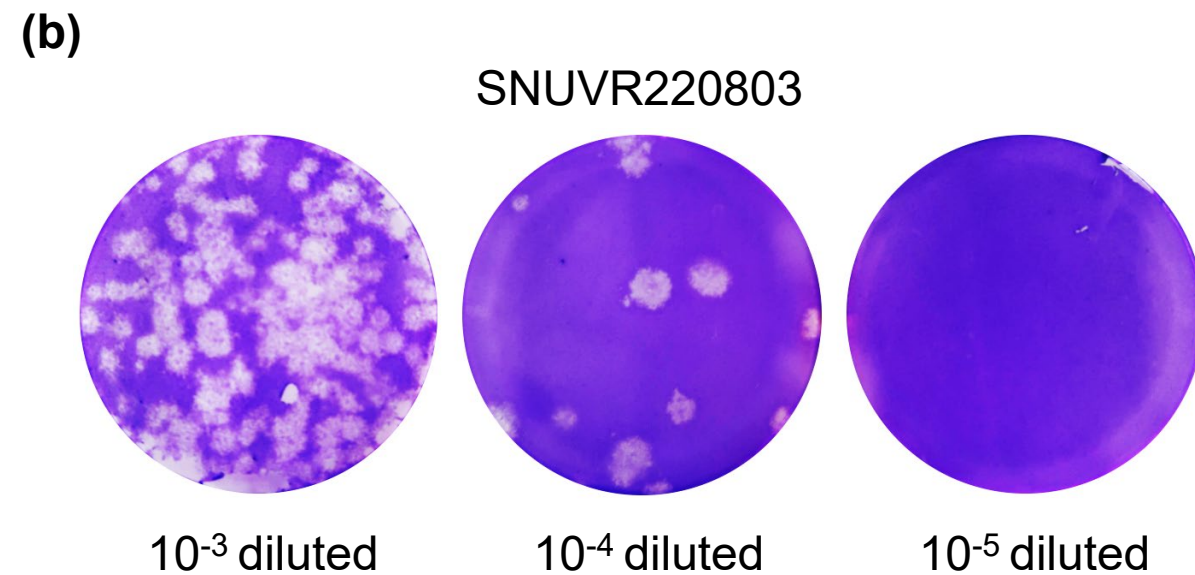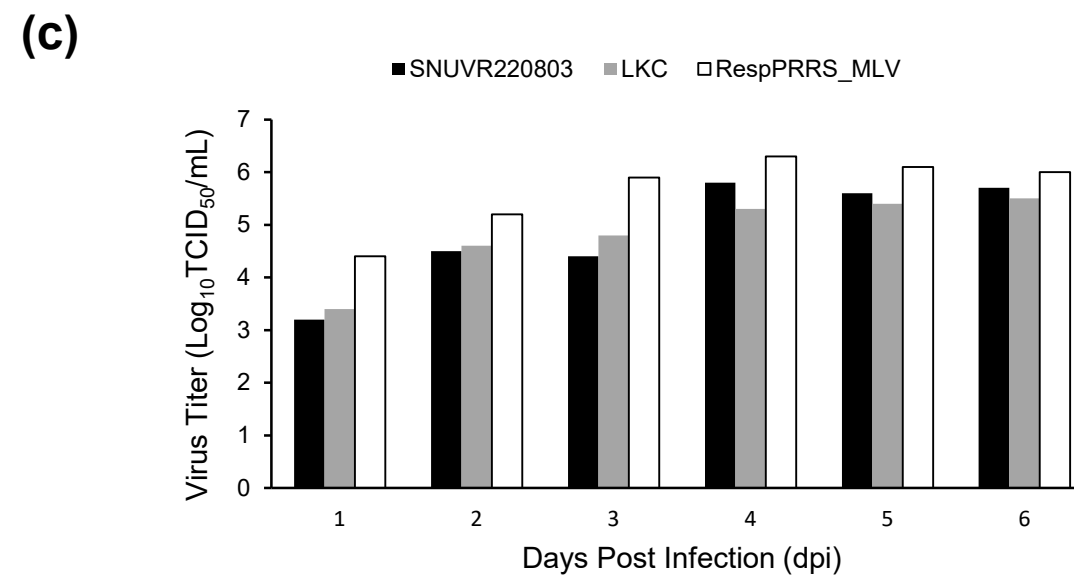

Supplement: Supplementary 2 — Figure 1: immunofluorescence assay (IFA), plaque assay, and virus titer results for SNUVR220803. (a) Shows the IFA results for PAM cells that were either mock-inoculated or inoculated with SNUVR220803. (b) Demonstrates plaque formation in MARC-145 cells inoculated with SNUVR220803. (c) Compares the virus titers of SNUVR220803, another LKC SNUVR240314 strain, and RespPRRS_MLV. [file 5785557.f2.pdf]
